# Yearning for movement. A qualitative interview study exploring physical activity in the context of endometriosis-associated chronic pain

**DOI:** 10.1177/17455057261455291

**Published:** 2026-05-21

**Authors:** Josefin Larsson, Birgit Heckemann, Emma Varkey, Axel Wolf, Sofia Jonsvik, Karin Sundfeldt, Paulin Andréll

**Affiliations:** 1 Department of Anaesthesiology and Intensive Care Medicine, Institute of Clinical Sciences, 70712 University of Gothenburg, Sahlgrenska Academy, Gothenburg, Sweden; 2 Department of Anaesthesiology, Intensive Care Medicine and Pain Medicine, Region Västra Götaland, 548809 Sahlgrenska University Hospital, Gothenburg, Sweden; 3 Institute of Health and Care Sciences, 70712 University of Gothenburg, Sahlgrenska Academy, Gothenburg, Sweden; 4 Department of Health and Rehabilitation/Physiotherapy, Institute of Neuroscience and Physiology, 70712 University of Gothenburg, Sahlgrenska Academy, Gothenburg, Sweden; 5 Department of Occupational Therapy and Physiotherapy, Region Västra Götaland, Sahlgrenska University Hospital, Gothenburg, Sweden; 6 University of Gothenburg Centre for Person-Centred Care (GPCC), Sahlgrenska Academy, University of Gothenburg, Gothenburg, Sweden; 7 Institute of Nursing and Health Promotion, Oslo Metropolitan University, Oslo, Norway; 8 Department of Obstetrics and Gynecology, Institute of Clinical Sciences, 70712 University of Gothenburg, Sahlgrenska Academy, Gothenburg, Sweden; 9 Department of Obstetrics and Gynecology, Region Västra Götaland, Sahlgrenska University Hospital/Sahlgrenska, Gothenburg, Sweden

**Keywords:** endometriosis, chronic pain, pain management, physical activity, lifestyle, qualitative research, person-centred care

## Abstract

**Background:**

Chronic pain is a common symptom of endometriosis with significant negative impact on physical and social activities. Patients perceive that healthcare professionals lack knowledge about the condition, and that available treatment options provide insufficient pain relief. Physical activity is recommended for endometriosis-associated pain in Swedish guidelines. However, little is known about how patients perceive and engage in physical activity in daily life.

**Objectives:**

To explore physical activity, self-management strategies and healthcare experiences in individuals with endometriosis-associated chronic pain.

**Design:**

A qualitative descriptive design.

**Methods:**

We conducted semi-structured individual interviews with 15 participants between December 2021 and September 2022. The interviews were audio-recorded, transcribed verbatim and analysed using qualitative content analysis.

**Results:**

The overarching theme identified from the analysis, “Yearning for movement”, captures both the strong desire to be physically active and the challenges of doing so while living with endometriosis-associated chronic pain. Three categories illuminate the various paths participants took in their efforts to manage physical activity. “*Monitoring and assessing*” describes participants’ continuous monitoring of their pain and energy levels and assessment of their ability to participate in social or physical activities. “*Seeking healthcare*” describes the participants’ needs for greater support and understanding from healthcare professionals. “*Adapting and trying to take action*” describes the actions the participants took to manage physical activity in daily living.

**Conclusion:**

Individuals living with endometriosis-associated chronic pain experience considerable limitations on physical functioning and a continuous time-consuming and laborious monitoring of pain and energy levels. Participants wished for increased knowledge about chronic pain and greater support for physical activity. These results call for improved support from healthcare for individuals suffering from endometriosis-associated chronic pain for both physical activity as well as pain-education.

## Background

Endometriosis is a chronic, gynaecologic and inflammatory disease in which endometrial-like tissue grows outside the uterine cavity, leading to inflammation and adhesions.^1,2^ Approximately 6-10% of women of reproductive age are affected globally, though prevalence varies by region and diagnostic criteria.^3,4^ Dysmenorrhoea is one of the most common symptoms of symptomatic endometriosis. Other common clinical manifestations include pelvic pain, dysuria, dyschezia and deep dyspareunia, as well as infertility and subfertility.^1,5,6^ The pain severity varies widely, ranging from asymptomatic to daily pain in multiple areas of the body.^2^ Hence, many individuals suffering from endometriosis also suffers from chronic pain, defined as persistent or recurrent pain lasting longer than three months.^7^ For many, endometriosis has a significant impact on quality of life adversely affecting physical activities, sexual health, social activities and personal relationships, contributing to reduced work productivity and to a considerable economic burden.^8–10^ Early diagnosis is crucial to prevent symptom and disease progression.^11^ However, diagnosis is often delayed by 5 to 12 years,^12^ due to multiple factors, including the heterogeneous presentation of symptoms, stigma surrounding symptoms, and limited access to specialist healthcare professionals.^2^ Although support and adequate information from healthcare professionals are crucial for successful self-management in chronic pain conditions,^13^ individuals with endometriosis often report unsatisfactory healthcare encounters.^14–16^ A 2018 Swedish report states that the normalising and trivialising of symptoms by healthcare professionals is common, often due to a lack of knowledge about the condition.^17^ The lack of knowledge about chronic pain is further highlighted in the review “An action plan: The Swedish healthcare pathway for adults with chronic pain”.^18^

Standard treatments for endometriosis include hormonal therapies and surgical interventions, which can be associated with complications.^19^ While the first-line analgesics is nonsteroidal anti-inflammatory drugs (NSAIDs),^20^ opioids may be used in cases of insufficient pain relief from NSAIDs.^21^ Many patients experience insufficient pain relief and side effects,^11,22^ with 60% reporting chronic pain.^23^ Due to the lack of effective treatment options, many turn to self-management strategies, such as dietary changes, heat, meditation, massage and rest,^24–26^ although evidence for their effectiveness varies.^14,27^ Physical activity (PA) is recommended as part of the management of chronic pain.^28^ In endometriosis, it has been suggested as a self-management strategy with effects on pain the day after exercise.^29^ Swedish national guidelines for chronic pain,^30^ as well as national guidelines and clinical recommendations for the management of pain in endometriosis, include physical activity among recommended approaches.^31,32^ The World Health Organisation (WHO) employs the original definition by Caspersen et al., describing PA as “any bodily movement produced by skeletal muscles that requires energy expenditure,”^33^ and further clarifies that PA includes movement undertaken during sport and leisure time, for transportation, at work, and in domestic settings.^34^ A Cochrane review has reported that PA improves function and may reduce pain in chronic pain patients.^35^ PA can also improve physical as well as psychological well-being.^28,35^ Given that many individuals with endometriosis experience substantial limitations in physical functioning and impaired mental health including depression and anxiety,^36^ the benefits of PA may be particularly relevant for this population, due to the dual effect of PA on both chronic pain and mental health, as anxiety and depression also modulate pain.^37^ However, two recent systematic reviews concluded that while PA could have positive effects on pain and well-being, the available studies are few, small and methodologically heterogeneous, indicating that more robust and larger-scale research is needed to determine the effectiveness of PA for endometriosis-associated pain.^38,39^

There is limited understanding of how individuals with endometriosis-associated chronic pain engage in PA and how pain influences their participation in PA. Furthermore, there is limited data on what support is provided from healthcare. A qualitative approach can provide deeper insights into patients’ experiences, perceptions, and strategies^40^ for managing physical activity in everyday life. To address this knowledge gap, the aim of the study was to explore physical activity, self-management strategies and healthcare experiences in individuals with endometriosis-associated chronic pain.

## Methods

### Design and setting

The manuscript conforms to the Consolidated criteria for reporting qualitative research (COREQ).^41^ For this qualitative descriptive interview study, participants were recruited from an ongoing multicentre randomised controlled trial (RCT) investigating the pain-relieving effect of transcutaneous electrical nerve stimulation (TENS). The RCT includes patients with endometriosis-associated chronic pain receiving treatment at two specialist gynaecology clinics in West Sweden: one at a University hospital and one at a county hospital (clinicaltrials.gov: NCT05152264; Västra Götaland Region (VGR) registry, www.researchweb.org/is/vgr; 274998). The inclusion criteria for the RCT were ≥18 years of age, endometriosis verified by laparoscopy or ultrasound, endometriosis-associated chronic pain, and stable (for at least three months) endometriosis-specific hormonal drug therapy. Exclusion criteria for the RCT were inadequate knowledge of the Swedish language, alcohol or substance abuse, pregnancy, pacemaker, malignant disease with limited life expectancy, decreased skin sensitivity, electro-acupuncture, severe untreated psychiatric disorders including psychiatric disease and/or psychological conditions which are the primary determinant to the patient’s pain condition, and utilisation of >90 morphine equivalents/day.

### Sample characteristics and recruitment

After giving consent to participate in the RCT, but before randomisation, the first 16 individuals were invited consecutively to take part in the present individual interview study. Of these, 15 agreed to participate and one declined. After consecutively including and conducting single interviews with 15 patients, and discussing reflections from field notes prior to each interview to assess whether new perspectives emerged in the data (JL and BH), we deemed the depth and breadth of the information to be sufficient to address the research questions.^42^

### Data collection

The first author (JL) conducted semi-structured individual interviews (n=15) via a digital care meeting service used by the care provider between December 2021 and September 2022. The interviews were recorded and ranged in duration from 35 minutes to 75 minutes (mean 53 min). All interviews were conducted in Swedish.

The interview guide (see Supplemental Material) was developed by authors JL, BH, EV and PA and tested for face validity in one pilot interview with a participant included in the RCT, prior to randomization. The pilot interview was recorded, transcribed and discussed within the researcher group. The interview guide was deemed suitable and not requiring further changes. The pilot interview was not included in the data analysis. The interview guide was used consistently across all interviews and started with a broad question about living with endometriosis, followed by more specific topic areas (Table 1).

**Table 1.** Topic areas and examples of questions in the interview guide.

Topic area	Example questions
Strategies	“What strategies do you have to manage chronic pain in your daily life?”
Physical activity	“Can you describe how your chronic pain affects your physical activity?”
Healthcare encounters	“Can you describe your experience of the treatment and care provided when you have sought help for endometriosis-associated pain?”

JL transcribed the first six interviews to familiarise herself with the content.^43,44^ The remaining interviews (n=9) were transcribed by a professional typist and subsequently checked against the audio recordings by the first author to ensure accuracy prior to analysis. All fifteen interviews were transcribed verbatim and in accordance with a transcription guide directly after the recording.^45^

### Analysis

Data were primarily analysed by the first author JL and the second author BH. JL is a doctoral student in medical science, nurse anaesthetist and pain nurse. JL is the project coordinator in the RCT with good knowledge in the research field, but limited prior experience with qualitative research; thus, she received training in qualitative methodology, including interview technique before data collection. JL had no prior or ongoing clinical relationship with the participants. BH, the second author, is a registered nurse, associate professor and senior researcher with significant experience in qualitative research.

We analysed the data using qualitative content analysis according to Graneheim and Lundman.^46^ For purposes of clarity, we describe this iterative process in five major steps. In the first step, the researchers (JL; BH) read and, using the interview guide for guidance, identified passages relevant to answering the research questions in a subsample of four transcripts.^47^ At this stage, we generously included text passages to ensure that we captured all relevant content. To mitigate the risk of introducing bias into our choice, we discussed each interview and text inclusion in depth during the process.^47^ JL and BH deemed four interviews a sufficiently varied and rich basis for developing an initial coding frame.^47^ In step two researchers (JL; BH) deductively identified and summarised meaning units (one or several sentences), using a preliminary coding framework derived from the interview guide. In step three, the coders (JL; BH) inductively recoded the subsample to capture the richness of the interviews and identified and defined additional codes and subcodes. In step four, two researchers (JL; BH) compared and discussed the subsample codes and their definitions to identify and eliminate overlaps and ambiguities, and to define a final coding framework, which, in step five, was applied to the entire sample. The final coding frame underwent further minor refinements and revisions to ensure it captured the richness of the material.

The coding process included one main coder (JL) guided by an experienced researcher (BH). As the coders coded free responses to open-ended interview questions and they did not work as independent raters, formal intercoder reliability coefficients such as Cohen’s kappa or Krippendorff’s alpha^48^ were not deemed meaningful for interpretation. However, we followed a negotiated agreement process^49^ by creating an audit trail of documentation of disagreements and their resolution to demonstrate rigour in the process. During the final coding round, 20% (n=94) of all coded units (n=469) were for flagged for discrepancy and discussed until agreement was reached. Finally, the categories, subcategories and their respective definitions were discussed and interpreted into a theme by a multiprofessional team with extensive experience in chronic pain treatment (PA; EV), nursing care (JL; SJ; BH) and qualitative research (BH; EV). Table 2 presents a schematic example of the data analysis process from meaning unit to category.

**Table 2.** The content analysis process.

Meaning unit	Code	Subcategory	Category
Exercise and movement often trigger pain. Which also means that I get scared when I have an inkling [about worsening pain] and don´t really know if it will go into a full attack or if it will stay as it is. So should I really go and work out now?	Impact on physical activity	Pain intensity	Monitoring and assessing
I wish there was, like, someone in my town who could have a physiotherapy group with training for endometriosis pain. So that there would have been some social aspects involved.	Healthcare encounters and care	Wishes and needs	Seeking healthcare
I have searched online, checked out forums, and joined the endometriosis patient association and other things like that. I have in fact learned more from outside the health care system.	Strategy	Self and peer	Adapting and trying to take action

The transcripts were managed with the Atlas Ti (version 23.07) software for computer-assisted qualitative data analysis during the coding step. In step 5, we transferred the meaning units to an Excel spreadsheet and used whiteboarding for the identification of themes, categories and subcategories, and the interpretation of the data.

### Ethical considerations

The study was approved by the Swedish Ethical Review Authority (ref. 2021-02958, June 6, 2021; amendment ref. 2022-05480-02, November 7, 2022). Participants received oral and written information about the study and provided written informed consent prior to the interviews. The study was conducted in accordance with the Declaration of Helsinki of 1975 as revised in 2013 and the Swedish National Good Research Practice guidelines.^50^ Given the sensitive nature of discussing experiences of endometriosis-associated chronic pain, participants were informed that they could decline to answer any question or withdraw from the interview at any time without providing a reason. Care was taken during the interviews to ensure that participants felt comfortable sharing their experiences.

### Reflexivity

The team had regular meetings to discuss the interview process, data analysis and interpretation to challenge and critically discuss the reading and interpretation of the material. As the research team consisted of clinicians and researchers with broad experience in chronic pain rehabilitation our analysis and interpretation are influenced by our collective clinical experience as nurses, physicians and physiotherapists. However, all members of our team are strongly committed to models of care that emphasise patients’ active involvement and partnership in care. This orientation guided our interest in the topic, the interview style and our reading of the material as it made us curious to discover both the patients’ limitations and resources in our attempts to understand the complexity of the study participants’ lives.

## Results

### Characteristics of the participants

The fifteen participants ranged in age from 24 to 46 and had experienced their pain for an average of 20 years (range 11-31). All participants were Swedish-born, the majority of participants had completed high school or had a higher education. However, the participants were in different life phases, including young and middle-aged adults, and some were parents. All participants had lived with endometriosis-associated chronic pain for >3 months (Table 3) and received stable endometriosis-specific hormonal drug therapy at the time of the interview.

**Table 3.** Background data for the 15 participants.

Age mean (range)	32 (24-46)
Time since pain debut mean years (range)	20 (11-31)
Pain mean daily pain intensity most recent week according to NRS* (range)	6.5 (1-10)
Education, highest completed	​
University degree	6
High school (12 years)	8
Primary school (9 years)	1
Current work status	​
Full-time employment	5
Part-time employment	4
Student	2
Full-time sick leave	4

*Numerical Rating Scale 0 = no pain and 10 = the worst imaginable pain.

### Findings

The analysis resulted in one overall theme – “Yearning for movement” – with three categories – “Monitoring and assessing”, “Seeking healthcare”, and “Adapting and trying to take action” – and eight subcategories, all of which are presented in Table 4. The subcategories encompass both physical activity and self-management strategies which we found to be closely intertwined.

**Table 4.** Theme, categories and subcategories.

Yearning for movement	
Category	Subcategory
Monitoring and assessing	Pain intensity
	Energy levels
Seeking healthcare	Professional consultation
	Wishes and needs
Adapting and trying to take action	Self and peer
	Striving for routines
	Shifting focus
	Accepting help

### Theme: Yearning for movement

The main theme captures the participants’ yearning for movement. A strong desire for PA was expressed as well as a persistent struggle toward this goal, despite the challenges that made it difficult to live the active life they desired.

### Category 1: Monitoring and assessing

The majority of participants described considerable limitations in their ability to engage in PA in daily life, such as during leisure time, sports, transportation, work, and domestic tasks. All described a need to continuously monitor their present or impending pain and energy levels and to assess whether PA was possible to carry out, or if it could lead to increased pain and fatigue. Their current pain and energy levels, as well as their uncertainty due to the day-to-day fluctuations, often hindered participants from performing PA. Pain and energy levels were often described as intertwined and interdependent.

#### Pain intensity

The pain described by the participants was ever-present in varying intensity. It was constant in its chronic form, but also in the form of relapses of acute and disabling pain, often with a sudden onset. In daily life, several frequently monitored their pain to gauge whether it was possible to engage in PA, and most participants expressed frustrations as well as a longing for enhanced ability to be physically active.

I want to be able to live like I did before I had pain every day, be able to exercise and do things with friends, ride a bike or whatever. Now I can’t do those things because I’m tired and in pain. And if I ever am active, I usually get pain after that. You have to think one step ahead – ‘What are the consequences if I do this?’ (IV4)

Monitoring of current pain was sometimes influenced by the fear of increased pain intensity.

Exercise and movement often trigger pain. Which also means that I get scared when I have an inkling [about worsening pain] and don´t really know if it will go into a full attack or if it will stay as it is. So should I really go and work out now? (IV13)

For several of the participants, this uncertainty resulted in avoidance of PA.

What’s so difficult is, you never know when they come, the pain attacks ... I don’t dare go out to certain places and so on, because of that. (IV14)

#### Energy levels

Most participants described daily life with endometriosis-associated chronic pain as one characterised by a great lack of energy. Several described frequently monitoring energy levels and how lack of energy impacted on their ability to be physically active.

You really become quite isolated, socially and everything. You can’t go out and do things [with friends]. I constantly have to think, can I afford to possibly trigger a pain flare? Do I have something important tomorrow that I’ll need my energy for? (IV13)

What I do is work, and then I don’t have the energy for anything else. I can manage my work, but everything beyond that … I see friends on Instagram being in the outdoors… All I think about is when I can go to bed. (IV 8)

### Category 2: Seeking healthcare

This category describes the participants’ experiences with healthcare professionals and perceived shortcomings in care provision. Some participants described positive healthcare encounters; however, the majority described dissatisfaction with the communication and care from healthcare professionals, as well as a general lack of guidance in PA and self-management.

#### Professional consultation

Many participants recalled that they had received treatment for endometriosis-associated chronic pain within the healthcare system for several years and their descriptions of the quality of their interactions with healthcare professionals ranged widely. Some shared positive experiences, describing feelings of being understood and taken seriously as well as receiving adequate information.

This one doctor has listened and taken in and has never trivialised [my pain] in the way that others have done. And has kind of tried to explain different things ... We have been a team, more or less. Instead of standing in separate corners. (IV 2)

However, most participants described predominantly negative experiences with long wait times before follow-up visits and encounters characterised by scant information regarding the disease, treatment options and possible self-management strategies other than analgesics.

It’s a chronic condition, something you’re supposed to learn to live with. But after I got the diagnosis, they just left me on my own. There’s no support. This is chronic … something I’ll have to live with for the rest of my life. How am I supposed to live with this? (IV 7)

A few participants had experience with physiotherapy; some had positive experiences, perceiving that it contributed to increased understanding of one’s own body.

Now that I’ve started seeing a physiotherapist, I’ve realised that chronic pain affects the whole body enormously. And I’ve kind of started to connect the dots, you know. (IV 11)

Others experienced that they lacked long-term follow-up as well as expert knowledge about endometriosis.

I booked an appointment with a physiotherapist, but I only went once, because I felt she didn’t understand endometriosis pain at all. To her, it was just, like, a bit of back pain. (IV 12)

#### Wishes and needs

The majority of participants emphasised how endometriosis affected most aspects of their lives and highlighted the need for more comprehensive and multiprofessional care. They desired professional support and coaching from multiple healthcare specialists and wished for information about the available support in the broadest sense, from pain medicine specialists, physiotherapists, counsellors, psychologists, dieticians or sexual counsellors.

It needs to be a multidisciplinary treatment, with counselling, physiotherapy, medication, all of it. It has to be a whole package. You need all those parts, because it [endometriosis] affects your whole body and your whole life. Treating just one thing isn’t enough. (IV4)

Most expressed a wish for more support from well-informed healthcare professionals, including improved continuity with a clearer care plan, regular follow-ups and evaluation of treatment.

I would have liked to have had a care plan. Like, ‘Let’s try this for three months and then we’ll have a follow-up to discuss how things are going, and if this [the plan] doesn’t work, then we can try this’. (IV 11)

Many wanted more extensive information regarding the disease and its management, suitable for their personal needs.

I would have liked to learn more about the pain. I would have liked more information about what help is available for endometriosis. More than just being prescribed painkillers. (IV12)

Several participants expressed a need for group support, facilitated by healthcare professionals, such as conversations with others living with endometriosis-associated pain and receiving physiotherapy.

I wish there was, like, someone in my town who could have a physiotherapy group with training for endometriosis pain. So that there would have been some social aspects involved. (IV 5)

### Category 3: Adapting and trying to take action

This category describes how the participants adapted and took certain actions to manage their pain and lack of energy in their efforts to manage PA in daily living. Several put into action the information they found online and recommendations from peers.

#### Self and peer

Most participants were actively seeking online information on their own, e.g., about the disease, its symptoms, hormonal or analgesic treatment options and alternatives to conventional treatments. The search for pain-relieving self-management strategies involved yoga for pelvic pain, podcasts describing breathing exercises and meditation and complementary treatments, such as TENS or acupuncture.

I have searched online, checked out forums, and joined the endometriosis patient association and other things like that. I have in fact learned more from outside the health care system. (IV8)

I've learned various tricks [from YouTube] on how to relax the pelvic floor. What circular movements to do and things like that. So I try that. (IV 4)

#### Striving for routines

Several participants expressed a desire for and pursuit of routines for PA, although, for many, such aims were continuously interrupted due to day-to-day fluctuations in pain and energy levels or relapses of acute pain and the need for rest.

I try to get in routines that I know I need, to get out and exercise. But once you start getting into the habit, the pain flares up and you end up stuck in bed. And then I lose all my routines again. You don’t have long periods when you don’t need to rest or need to lie down and cope with the pain ... I always have to, like, ‘energy-budget’ for rest. Which makes it so difficult to do things. (IV13)

#### Shifting focus

While most of the participants expressed great challenges in terms of PA, for some, in periods of more moderate pain, it could be an efficient way to distract themselves and a strategy to manage pain. Yoga, going for walks or lifting light weights could help shift their focus away from the pain.

I do try to keep up with the strength training, but I skip it when it [the pain] is really bad. Then it’s just not possible. But I think it works for my everyday pain, it relieves the pain a bit. Then it becomes more like, ‘okay, I’m here in the moment, and this is what we need to focus on right now. (IV 2)

Several participants also described frustration regarding the fact that pain frequently interrupted their current activity, not just PA, and it was necessary to shift focus from the activity to manage the pain, sometimes pushing relentlessly through to accomplish something.

I worked so hard even though I was in pain. I went to work and ended up vomiting. And then I cried, and then I vomited some more, and then I cried even more. But still, I wanted to work. (IV7)

#### Accepting help

All participants agreed that they were, at times, in need of help from family members or friends with managing daily PA such as household chores, grocery shopping and cleaning.

*Recently, I’ve been having more and more difficulty, like when I go shopping, I struggle to carry my groceries home because it’s so painful. Sometimes it feels like my uterus is going to explode. So my partner has taken all that on, I am not carrying anything. (IV12*)

The participants who were parents described how they often depended on help to take care of their children.

*He [partner] takes care of everything with the children. He stays home from work if necessary. He takes the children on trips so I can rest. He arranges for a babysitter if he cannot be at home*. *(IV 6)*

## Discussion

This qualitative individual interview study aimed to explore physical activity, self-management strategies and healthcare experiences in individuals with endometriosis-associated chronic pain. Our main theme, “Yearning for movement”, illustrates the participants’ desire for increased PA as well as their persistent struggle to live a physically active life, despite the challenges. The contribution of this study is two-fold. First, it highlights the importance and challenge of PA in individuals with endometriosis-associated chronic pain, and second, it identifies a gap between patients’ needs and current healthcare provision.

PA is a recommended self-management for endometriosis,^29^ yet previous research has shown that the condition limits the ability to be physically active.^51^ Our study further elucidates how individuals with endometriosis-associated chronic pain perceive and engage in PA. The participants’ lives revolved around time-intensive, endless and careful monitoring of pain and energy levels and assessment of their ability to PA, as described in the category “Monitoring and assessing”. Fear of triggering increased pain and uncertainty due to day-to-day pain and energy fluctuations often resulted in the avoidance of PA. This fear-related avoidance among individuals with other chronic pain conditions is well recognised and described by Vlaeyen and Linton in the fear-avoidance model, in which an expected increase in pain leads to hesitancy and avoidance of PA. The consequences of this avoidance behaviour include an increased risk of withdrawal and reinforcing mood disturbances, such as frustration and depression, which are known to be associated with decreased pain tolerance.^52^ From a clinical perspective, it is important to recognise fear-related avoidance in the rehabilitation of patients with endometriosis-associated chronic pain. PA must be adapted to an individual’s ability and gradually increased depending on the underlying pain mechanisms, to minimise the risk of pain flare-ups and to increase self-efficacy.^53,54^ Self-efficacy refers to an individual’s belief in their ability to perform actions required to achieve desired outcomes.^55,56^ In a health-related context, this includes individuals’ beliefs in their ability to influence their health and perform health-related actions.^57^ Higher self-efficacy has been associated with improved physical and mental quality of life among individuals with endometriosis.^58^ Furthermore, Lööf and Johansson found in an interview study that pain anticipation and fear‐avoidance beliefs regarding PA could affect behaviour and increase patients’ focus on their fragile physical and emotional state, leading to a mistrust of their body’s energy levels and physical capacity. This is described as a maladaptive body awareness, involving increased attention placed on the uncomfortable body. Rehabilitation of chronic pain may therefore benefit from helping patients develop more adaptive body awareness and trust in their bodies.^59^ Tennfjord et al. have described how pain management education and supervised group- and individualized exercise training in endometriosis contributes to reduce fear amongst the women when exposed to various intensities and types of exercises in a safe environment with professional supervision.^60^

Avoidance of PA seemed to coexist with overactivity among the participants. Overactivity has been previously described in chronic pain patients. These individuals are more prone to ignoring or distracting themselves from their high levels of pain, leading to severe pain aggravation and periods of incapacitation during which they are unable to carry out activities of daily living.^61,62^ Individual variations in the relationships between pain interference and psychological processes in chronic pain have been highlighted by Sundström et al. The authors argued that there is a lack of correspondence between group-level and individual-level findings, emphasising a shift toward more person-centred approaches for managing chronic pain.^63^

Swedish national guidelines recommend PA for chronic pain conditions and endometriosis.^30,31^ Despite this, in the category “Seeking healthcare”, the participants stated they need increased support from and interactions with physiotherapists with expertise in the field, to help them improve their physical functioning. This desire is also apparent in previous research involving patients with other chronic pain conditions, which has also shown that PA is greatly valued and desirable even though performance can be difficult.^53,54^ In addition, the participants expressed their wishes and needs to receive professional support and guidance from various healthcare professionals with expert knowledge about endometriosis-associated pain. They frequently referred to a need for advice and support from pain specialists as well as from counsellors, psychologists, dieticians, and sexual health counsellors. Moreover, Swedish national guidelines recommend a multiprofessional collaboration for patients with chronic and complicated pain conditions to capture the full complexity of the condition.^31^ The importance of a multi-disciplinary care setting has also been advocated in previous research concerning individuals affected by endometriosis.^64–68^

Our result further highlights the participants’ wishes and needs for better communication with healthcare professionals, involving clearer care plans with thorough follow-ups and better information regarding chronic pain, PA and self-management. These results support the need for pain education, including management and lifestyle strategies for patients in endometriosis care. This requires healthcare professionals to be well informed and have good knowledge of chronic pain and its management, an area currently underdeveloped in Swedish healthcare and in need of greater prioritisation.^18^ The benefits of pain-education in endometriosis has been shown by Kvåle et al., following a pain-management course, participants reported increased ability to self-manage endometriosis and demonstrated a progression from passive to active participation in their own care.^69^

Insufficient communication and lack of information have been identified in previous studies.^14,15,64,70^ An evaluation of the Swedish National Guidelines for Endometriosis Care from 2019, described an ongoing lack of knowledge among healthcare professionals and a widespread lack of follow-up of prescribed treatments.^71^ Our study affirms that this issue is still relevant and is highly problematic, not least in light of Swedish healthcare laws, stipulating person-centred care and active participation of patients.^72^ In person-centred care, the patient is acknowledged as a capable and equal partner and as an expert in both the development and assessment of care. The core component of person-centred care is partnership, grounded in shared decision-making and documentation, between the patient, their relatives, and healthcare professionals and entails working with the patient’s resources, needs, wishes and goals.^73^ Self-management is considered to play an important role in person-centred care by co-creating a care plan that supports the patients to take control over their own health.^73,74^ The present study indicates that the implementation of person-centred care in healthcare for individuals suffering from endometriosis-associated chronic pain seems to be lacking, from the perspective of patients, and it highlights specific challenges in using PA as an effective self-management strategy. Previous research from Werner et al. shows that one of the primary reasons for not utilizing self-management strategies are lack of information and costs for the individual.^75^ Healthcare professional led physiotherapy is subsidised the Swedish healthcare system, and still our participants perceived they lacked information regarding available support for increased PA.

Lastly, the category “Adapting and trying to take action” further highlight the participants’ proactivity in their search for help and understanding. This desire for pain-management strategies other than analgesics, and the common use of online resources to find such strategies, indicate that healthcare services need to better support this patient group through evidence-based (online) resources, to provide them with accurate information and to assist them in managing their daily pain. Pavic et al. have described the value of digital-technology based interventions for supporting individuals suffering from endometriosis and highlight the importance of a methodological framework to structure their development to optimise patient support.^76^ As advocated in person-centred care, such an approach could enable a partnership that would encourage individuals to actively take part in finding solutions to their problems rather than being a passive target of medical intervention.^73^

### Clinical implications

The participants in our study found the care provided by the healthcare system to be insufficient. Coxon et al. reason that modern endometriosis management should be personalised, and pain management should be based on a multidisciplinary treatment approach, in which patients’ symptoms and expressed needs are acknowledged by healthcare professionals.^77^ To manage a life with endometriosis-associated chronic pain, it is crucial that patients are given the opportunity to play an active role in their care plans.^14^ A person-centred collaboration between patients and healthcare professionals is needed to facilitate the development of suitable care plans that include support to increase an individual’s ability to engage in PA.

Our participants wished for education regarding chronic pain, self-management strategies and support for being physically active. From the participants’ perspective, there is an apparent lack of knowledge of endometriosis-associated chronic pain among healthcare professionals.^18^ Hence, there is a need for education focusing on endometriosis-associated chronic pain for both patients and healthcare professionals. Patients need guidance from healthcare professionals to implement evidence-based self-management strategies to reduce their time- and energy-consuming monitoring of their pain, a matter that warrants further research.

### Study strengths and limitations

Qualitative methods are suitable for exploring experiences, perceptions, and behaviour, and provides deeper insights into real-world problems.^78^ The qualitative interviews were hence an appropriate method to answer the research questions, as it provided deep insights of how the participants perceive and engage in PA. Our preunderstanding was informed by the literature, entailing that endometriosis-associated chronic pain can be associated with decreased ability to be physically active. The participants were asked how their chronic pain affects PA, which can be considered as a leading question. However, the interview technique with open ended questions gave the possibility to nuanced answers. Participants in the present study were recruited consecutively from an ongoing RCT in which eligible individuals were identified by midwives based on the inclusion and exclusion criteria. While this ensures diagnostic clarity in the RCT, we recognize that broader inclusion criteria, including individuals with undiagnosed chronic pelvic pain, could have captured a wider range of experiences of PA and support from healthcare. We did not exclude individuals with other chronic pain comorbidities, in terms of irritable bowel syndrome, ulcerative colitis and fibromyalgia, although these may affect endometriosis-associated pain intensity. However, since these comorbidities are common in individuals with endometriosis,^79,80^ the participants can be considered representative for the patients in Swedish endometriosis specialist care.

The participants were aware of the researcher’s motivations for conducting this study, i.e. to improve care for patients with endometriosis. We cannot exclude the possibility that the participants decided to take part in the study because of their interest in the research. However, the main incentive for participation was most likely to try TENS in the RCT, as this interview study is part of a larger project evaluating the effects of TENS for endometriosis-associated chronic pain. In that sense, the participants might be a selected population interested in self-management strategies for their chronic pain, including PA.

During the interview process and data analysis, the entire research group was involved in discussing the material, which was considered rich, and involved diverse narratives. The research group analysing and interpreting the data consisted of a multidisciplinary team of healthcare professionals. The interpretation was therefore carried out through different professional lenses, increasing the relevance of the findings across healthcare professions. In the planning of the overarching RCT study the Swedish endometriosis patient association was consulted to ensure that the research question was relevant to individuals suffering from endometriosis and that the study design of the RCT was relevant. However, no patient representative was involved in the present study.

A further limitation is that the study took part in a Swedish healthcare context and only individuals who spoke fluent Swedish were included in the study. However, the participants’ descriptions represent a human experience of chronic pain and its influence on PA, which is independent of the healthcare system of the country in which one lives. The sample consisted of participants who had been referred to an endometriosis centre, although the participants described experiences that were acquired from various healthcare settings, which strengthens the transferability of the results.

## Conclusion

Individuals living with endometriosis-associated chronic pain experience considerable limitations on physical functioning and engage in continuous time- and energy-consuming monitoring of pain and energy levels in the assessment of their ability to perform PA. The yearning for a greater ability to engage in physical activities was clearly expressed. Participants wished for self-management strategies other than analgesics, increased knowledge about chronic pain and increased support for PA in multiprofessional care settings. Being physically active was described as intertwined with many aspects of life and was considered necessary to prevent withdrawal from social activities that lead to a better quality of life. These results call for increased support for self-management including PA as well as pain-education for individuals suffering from endometriosis-associated chronic pain, which in turn requires increased knowledge in this field for healthcare professionals. Further studies are needed to evaluate a multiprofessional person-centred care approach in patients with endometriosis-associated chronic pain.

## Supplemental material

Supplemental material - Yearning for movement. A qualitative interview study exploring physical activity in the context of endometriosis-associated chronic pain Supplemental material for Yearning for movement. A qualitative interview study exploring physical activity in the context of endometriosis-associated chronic pain by Josefin Larsson, Birgit Heckemann, Emma Varkey, Axel Wolf, Sofia Jonsvik, Karin Sundfeldt, and Paulin Andréll in Women's Health.

## Data Availability

The datasets generated and/or analysed during the current study are not publicly available due to ethical concerns (the study participants did not give written consent to share their data publicly).*
