# Supplementary material for: Yearning for movement. A qualitative interview study exploring physical activity in the context of endometriosis-associated chronic pain
Source: Womens Health (Lond). 2026 May 21;22:17455057261455291. doi: 10.1177/17455057261455291 (PMC13195239; doi:10.1177/17455057261455291)
Supplement: Supplemental material - Yearning for movement. A qualitative interview study exploring physical activity in the context of endometriosis-associated chronic pain [file sj-pdf-1-whe-10.1177_17455057261455291.pdf]

Interview guide

Opening question

☐ "Can you tell me about your experiences of living with endometriosis-associated chronic pain?"

Following questions

☐ "What strategies do you have for managing your chronic pain in everyday life?"

☐ "Can you describe how your chronic pain affects your ability to be physically active?"

By physical activity, we mean both activities in your everyday life that involve using your body, such as walking to work or school, gardening, doing household chores, playing with children, and activities performed with the purpose of exercise or training.

☐ "What role do your partner, family, friends, or other close persons play in your daily life with chronic pain?"

☐ "How do you experience healthcare professionals' attitudes and the care you receive when seeking help for endometriosis-associated pain?"

Closing

☐ Ask the interview participant to summarise the reflections, statements, or questions that were most important to them.

After the interview: notes and reflections

(e.g. How did the interview proceed? What was the atmosphere like? Did all questions work as intended? Are there any new aspects that should be explored further?)
